# Antagonistic Yeasts: A Promising Alternative to Chemical Fungicides for Controlling Postharvest Decay of Fruit

**DOI:** 10.3390/jof6030158

**Published:** 2020-08-31

**Authors:** Xiaokang Zhang, Boqiang Li, Zhanquan Zhang, Yong Chen, Shiping Tian

**Affiliations:** 1 Key Laboratory of Plant Resources, Institute of Botany, Innovative Academy of Seed Design, Chinese Academy of Sciences, Beijing 100093, China; zhangxk@ibcas.ac.cn (X.Z.); bqli@ibcas.ac.cn (B.L.); zhangzhanquan82@ibcas.ac.cn (Z.Z.); chenyong@ibcas.ac.cn (Y.C.); 2 College of Life Sciences, University of Chinese Academy of Sciences, Beijing 100049, China

**Keywords:** yeast, biological control, postharvest decay, fruit

## Abstract

Fruit plays an important role in human diet. Whereas, fungal pathogens cause huge losses of fruit during storage and transportation, abuse of chemical fungicides leads to serious environmental pollution and endangers human health. Antagonistic yeasts (also known as biocontrol yeasts) are promising substitutes for chemical fungicides in the control of postharvest decay owing to their widespread distribution, antagonistic ability, environmentally friendly nature, and safety for humans. Over the past few decades, the biocontrol mechanisms of antagonistic yeasts have been extensively studied, such as nutrition and space competition, mycoparasitism, and induction of host resistance. Moreover, combination of antagonistic yeasts with other agents or treatments were developed to improve the biocontrol efficacy. Several antagonistic yeasts are used commercially. In this review, the application of antagonistic yeasts for postharvest decay control is summarized, including the antagonistic yeast species and sources, antagonistic mechanisms, commercial applications, and efficacy improvement. Issues requiring further study are also discussed.

## 1. Introduction

As an important part of the human diet, fruit provides the body with beneficial vitamins, minerals, organic acids, and antioxidants. Fruits have been shown to have many health-related effects, such as anti-cancer effects, skin protecting effects, and postponing of senescence [1,2,3,4]. As orchards are usually far away from urban areas, and the fruit maturity occurs in a relatively short period, leading to a disparity between supply and demand in the market, which necessitates a certain period of storage and transportation to adjust for this disparity. However, postharvest spoilage, which involves rot, nutrient loss, and water content loss, occurs most often during storage and transportation, which leads to considerable economic losses. It has been reported that about 25% of total fruit production is wasted after harvest in developed countries, and the postharvest losses in developing countries account for >50% of total fruit production because of lack of efficient transportation and refrigeration facilities [5].

Fungi are the main cause of postharvest spoilage. Fruit rot can be induced by wound generated during harvesting, packaging, storage, and transportation, as well as the favorable growth conditions for pathogens (e.g., high water and nutrient content, low pH, and decreased resistance after harvest) [6]. During the process of infection, many fungi produce mycotoxins, which may enter the food chain via fresh and processed fruit products and then endanger human health. For example, *Penicillium expansum*, which causes blue mold in many fruits, leads to not only fruit decay but also the contamination of patulin, a teratogenic, carcinogenic, and immunotoxic mycotoxin [7]. Chemical fungicides have long been used to control postharvest decay. However, overdependence on traditional chemical fungicides has resulted in a variety of problems, such as fungicide residues, environmental pollution, and increased pathogen resistance to fungicides. Therefore, identifying safe and effective approaches to control postharvest fungal disease is urgent.

Since Gutter and Littauer first reported the use of *Bacillus subtilis* to combat citrus fruit pathogens in 1953, the biocontrol capability of microorganisms against postharvest decay has attracted widespread attention [8,9]. Among the various microbial antagonists, yeast and yeast-like fungi occupy an important position as they are environmentally friendly, exhibit good biocontrol efficacy against pathogens, possess adequate stress tolerance, and can potentially be genetically improved; additionally, there is a well-developed system for culturing, fermentation, storage, and handling of these antagonistic yeasts [10]. Moreover, yeasts have been used in food and beverage production for thousands of years and currently play an important role in the food industry. Thus, the utilization of yeasts is generally considered safe, and easily acceptable by market. With the great properties and application superiority, antagonistic yeasts are considered as a promising alternate to synthetic chemical fungicides [5,9]. Over the past few decades, great progresses have been made in biological control based on antagonistic yeasts, including strain isolation and screening, mode of action, improvement of biocontrol efficacy, and formulation. Particularly, several antagonistic yeasts with excellent biocontrol performance have been developed and registered as commercial products. Nonetheless, the widespread use of yeast antagonists to manage postharvest diseases still faces many challenges. A deeper understanding of the mode of action of antagonistic yeasts in postharvest biocontrol system is still needed; the inconsistency of performance of antagonistic yeasts under commercial conditions need be overcome; the market penetration of products is difficult.

Here, a comprehensive overview of the applications of antagonistic yeasts in postharvest decay control is presented, including the features of antagonistic yeasts, antagonistic mechanisms, efficacy improvement, and commercial applications. The latest research results are highlighted, and issues requiring further study are also discussed.

## 2. Features of Antagonistic Yeasts

Yeasts are a group of eukaryotic fungi, most of which are unicellular and reproduce by budding [11]. There are also a variety of phylogenetically different groups of yeast-like fungi, such as *Aureobasidium pullulans*. Antagonistic yeasts (also known as biocontrol yeasts) refers to yeast or yeast-like fungi that can inhibit or interfere with the growth, development, reproduction, or activity of phytopathogens. Wilson and Wisniewski summarized the criteria for the selection of ideal biocontrol agents in 1989 [12]. With the extensive research on antagonistic yeasts, the screening criteria for antagonistic yeasts have gradually improved [13]. An ideal antagonistic yeast should be genetically stable, have simple nutrient requirements, be effective in adverse environmental conditions and at low concentrations, and be effective against multiple fungal pathogens on various fruits [6,9]. Moreover, an antagonistic yeast should have favorable commercial potential: It should be able to grow on an inexpensive growth medium, be easy to store and dispense, and be compatible with other physical and chemical treatments (e.g., controlled atmosphere, low/high temperature, chemical fungicides/pesticides, and phytohormones [5]. As for biosafety, a desirable antagonistic yeast would be environmentally friendly, have no pathogenicity regarding the host fruits, produce no metabolites that are harmful to humans, and be unable to cause infection in humans [5,9].

**Figure 1:**
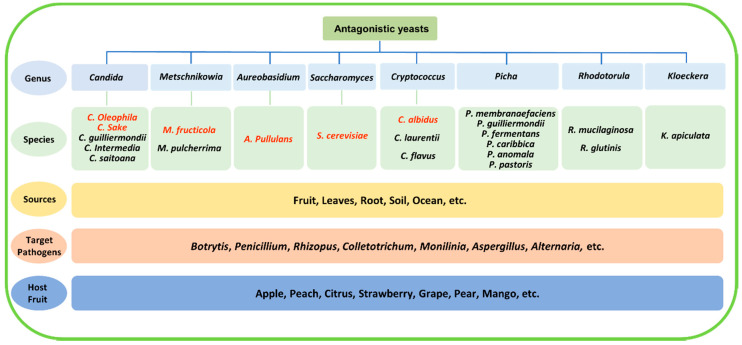
Representative antagonistic yeasts from various sources used for the management of postharvest decay. Species that have been already in commercial use are highlighted in red.

The isolation and screening process is the first step in the development of a biocontrol agent. Most antagonistic yeasts were isolated directly from fruit surfaces [14,15], but they have a wider distribution in nature, such as on leaves and roots and in seawater and soil (even Antarctic soil) [16,17,18,19,20]. So far, a large number of antagonistic yeasts have been isolated and screened. Some of them have been widely studied, such as *Candida* spp., *Cryptococcus* spp., *Metschnikowia* spp., *Pichia* spp., *Rhodotorula* spp., and yeast-like fungus *A. pullulans*, and several species, such as *Candida oleophila*, *Candida sake*, *Metschnikowia fructicola*, *A. pullulans*, *Saccharomyces cerevisiae*, and *Cryptococcus albidus*, have been developed as commercial products [21,22,23,24,25,26,27,28]. They have been demonstrated to antagonize common postharvest pathogens, including *Botrytis cinerea*, *Penicillium* spp., *Rhizopus stolonifer*, *Colletotrichum* spp., *Monilinia fructicola, Alternaria alternata*, and *Aspergillus niger*. Representative antagonistic yeasts that were isolated from various sources and are used for the management of postharvest diseases are shown in Figure 1.

## 3. Mechanisms of Action

**Figure 2:**
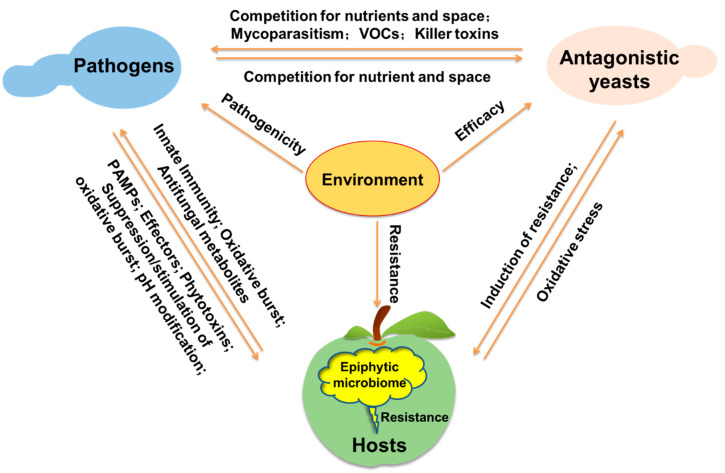
Schematic diagram of the possible interactions among components of the biocontrol system, including the pathogens, antagonistic yeasts, host, epiphytic microbiome, and environment. Antagonistic yeasts can inhibit pathogens through competition for nutrient and space, mycoparasitism, VOCs, and killer toxins. Conversely, pathogens also compete with antagonistic yeasts for nutrient and space to affect their colonization and growth. In addition, antagonistic yeasts can induce the resistance of hosts to inhibit infection, while reactive oxygen species (ROS) produced by hosts may be an oxidative stress to yeasts. During the interaction between fruit hosts and pathogens, hosts can resist the pathogen attack through oxidative burst, innate immune system, and antifungal metabolites, while pathogens can suppress host resistance through pathogen-associated molecular patterns (PAMPs), effectors, phytotoxins, pH modification, and suppression or stimulation of the oxidative burst. The epiphytic microbiome on hosts is also associated with the host resistance. Moreover, environmental conditions have a wide influence on the pathogenicity of pathogens, the efficacy of antagonistic yeasts, and the resistance of hosts.

Elucidating the mechanisms of action is the foundation for the development and application of antagonistic yeasts [29]. Compared with the impressive results achieved regarding the identification of antagonistic yeasts, the study of their mechanisms of action is relatively slow due to the complexity of the postharvest biocontrol system. In this system, the antagonistic yeasts, pathogenic fungi, and fruit hosts interact with each other under the influence of the environment, and the influence of the epiphytic microbiome should also be taken into consideration (Figure 2) [12,29,30,31].

The antagonistic yeasts are likely to function via multiple mechanisms, including competition for nutrients and space, mycoparasitism, induction of host resistance, production of volatile organic compounds (VOCs), and toxins [9,10,31]. With the increase in the number of annotated yeast genomes and the development of “omics” technologies and transformation technologies, the modes of action of antagonistic yeasts will be further deciphered in the near future [32,33,34].

### 3.1. Competition for Nutrients and Space

Both postharvest pathogens and antagonistic yeasts require nutrients (e.g., carbohydrates and nitrogen) and space to colonize and develop. Therefore, the competition for nutrients and space has been considered the primary mode by which antagonistic yeasts suppress postharvest fungal pathogens [5,29]. Once the antagonistic yeasts come into contact with the surface of the injured fruit, they will occupy the wounds and rapidly deplete the nutrients, which limits the germination of fungal spores [35,36]. After that, other mechanisms of action (besides the competition for nutrition and space) cooperatively come into operation to control the postharvest pathogens [8].

Carbon, nitrogen, and iron ions are the main nutrients needed for the growth of microbes. Compared with carbohydrates, nitrogen is considered to be a key factor limiting the growth of postharvest fruit pathogens, because most fruits are rich in sugar but limited in nitrogen sources such as amino acids. The application of exogenous amino acids reduced the antagonistic effect of the yeast *A. pullulans* against *Penicillium expansum* on apple fruit, indicating the importance of nitrogen competition to biocontrol efficacy [37]. Moreover, iron plays a crucial role in the growth and virulence of pathogens. Iron is a component of cytochromes, other heme proteins, and non-heme proteins; it is also a cofactor of various enzymes in fungal cells [9,38]. The yeast *Metschnikowia pulcherrima* can produce iron chelators to compete for the iron required by pathogens, thus strongly inhibiting the growth of the pathogens [39]. Parafati et al. also proposed that the consumption of iron ions plays an important role in the biocontrol effect of *M. pulcherrima* [40]. Some antagonistic yeasts can also produce siderophores to compete for iron in a low-iron microenvironment, thus inhibiting the germination and growth of pathogens. For example, rhodotorulic acid is a dihydroxamate siderophore produced by *Rhodotorula glutinis* that improves the biocontrol against *P. expansum* [41]. Siderophores produced by *A. pullulans* plays an important role in yeast growth and pathogen inhibition under iron deficiency environment [13,42].

Biofilms are dense microbial communities attached on fruit surfaces, and encapsulated by polymeric extracellular matrix (ECM) [43]. Formation of biofilm is considered as another strategy utilized by antagonistic yeasts to compete for space and nutrient [10,44]. Scherm et al. found that the biofilm formation of *S. cerevisiae* M25 was directly related to its biocontrol effect, with only the *S. cerevisiae* cells collected during the biofilm formation phase effectively controlling *P. expansum* on apples [45]. Biofilm formation has also been hypothesized to be a key mechanism of action of *Metschnikowia citriensis* against *Penicillium digitatum* and *Penicillium italicum* on citrus fruit [46]. Notably, it was reported that *Pichia fermentans* formed biofilms and inhibited postharvest decay in apple fruits but caused rapid decay in peach fruits in the absence of a plant pathogen [47], indicating the potential risk of dimorphic antagonistic yeast becoming pathogens.

### 3.2. Mycoparasitism

Mycoparasitism refers to the phenomenon of antagonistic yeasts feeding on fungal pathogens via attaching to the fungal pathogen hyphae and then secreting cell wall-degrading enzymes to destroy or lyse the fungal structures. Especially in the case of nutritional deficiencies, antagonistic yeasts tend to absorb nutrients from pathogenic cells, leading to the death of these “prey” cells. During mycoparasitism, a variety of enzymes are involved in the degradation of the fungal pathogen cell wall, especially β-1,3-glucanase (GLU), chitinase (CHT), and proteases [29], and these secreted enzymes are thought to play an important role in biocontrol [48]. Wisniewski et al. first reported the mycoparasitism of *Pichia guilliermondii* [49]. They observed that the yeast strongly adhered to the *B. cinerea* mycelium and caused hyphal collapse, which was presumably due to a lectin-like interaction. It has also been reported that both *Pichia membranefaciens* and *C. albidus* can attach to and degrade the hyphae of *P. expansum, M. fructicola*, and *R. stolonifer* [50]. Banani et al. found that the chitinase gene, *MfChi*, of the yeast *M. fructicola* was significantly induced by cell wall of the postharvest pathogen *M. fructicola*, and MfChi-overexpressing *Pichia pastoris* inhibited the brown rot of peach fruits [51]. In *C. oleophila*, GLU was demonstrated to be associated with inhibiting conidial germination and hyphal growth of *P. expansum* [52].

### 3.3. Induction of Host Resistance

Induction of host resistance, as one of the major mechanism of antagonistic yeasts for postharvest decay control in fruits, has also been extensively studied [53,54]. Antagonistic yeasts have been reported to act as biological elicitors in the interactions with fruit hosts [29,48]. Treatment with antagonistic yeasts can increase the expression of defense-related genes and enhance the activities of defense-related enzymes. Strongly induced activities of defense-related enzymes, such as CHT, GLU, phenylalanine ammonia-lyase (PAL), and peroxidase (POD), have been reported to be responsible for the biocontrol efficacy of *Cryptococcus laurentii* on postharvest decay caused by *A. alternata*, *M. fructicola*, and *P. expansum* [55,56,57]. Chan et al. found that the antagonistic yeast *P. membranaefaciens* could induce the activities of three pathogenesis-related (PR) proteins, which may contribute to the resistance improvement of peach fruit to *P. expansum* [33]. Similarly, induced expression of defense-related genes and the activities of defense-related enzymes by *W. anomalus* were considered as one of the possible mechanisms in inhibiting blue mold decay caused by *P. expansum* in pears [58].

Moreover, application of antagonistic yeasts can enhance activity of antioxidant enzymes, which may alleviate oxidative damage cause by reactive oxygen species (ROS) produced by hosts in response to pathogen infection. *P. membranaefaciens* has been reported to affect the activities of antioxidant enzymes, including POD, catalase (CAT), glutathione peroxidase (GPX), superoxide dismutase (SOD), and polyphenol oxidase (PPO), in peaches and sweet cherry fruits after inoculation with *P. expansum* [33,59]. Additionally, four antagonistic yeasts (*P. membranaefaciens*, *C. laurentii*, *Candida guilliermondii*, and *R. glutinis*) have been reported to increase the activities of POD and CAT, upregulate the expression of the corresponding genes, and reduce the levels of protein carbonylation in peach fruits caused by *M. fructicola* [60].

Antagonistic yeasts can also induce changes in secondary metabolites and cell structure related to disease resistance. Droby et al. found that the application of *C. oleophila* increased the levels of the phytoalexins umbelliferone, scoparone, and scopoletin in grape fruit peels [61]. El-Ghaouth et al. found that the antagonistic yeast *Candida saitoana* could induce host cell deformation, generate mastoid structures, and consequently inhibit *B. cinerea* infection [62].

Multiple mechanisms may be simultaneously involved in the resistance induction by antagonistic yeasts. For example, several antagonistic yeasts, such as *C. laurentii* [63], *P. membranaefaciens* [33], *P. guilliermondii* [64], *R. glutinis* [60], and *R. paludigenum* [25], induced changes in activities of both defense-related enzymes and antioxidant enzymes in fruit. Induction of disease resistance by antagonistic yeasts is also affected by pathogens and environmental conditions. As shown in Figure 2, there are complex interactions between the hosts, pathogens, antagonistic yeasts, and environment, which remains to be elucidated.

### 3.4. Production of VOCs and Killer Toxins

Compared to filamentous fungi, yeasts have a lower secretory capacity and produce only few secondary metabolites. Nevertheless, VOCs and killer toxins are metabolites that have been reported to exhibit antifungal activity.

VOCs are volatile compounds with low molecular weight (<300 Da), low polarity, and high vapor pressure. Some antagonistic yeasts can produce VOCs, and the mixture of VOCs has been proposed to play an important role in the control of postharvest pathogens under airtight conditions [48,65]. It was reported that *Candida intermedia* 410 inhibited the growth of *B. cinerea* on strawberries by releasing VOCs without direct contact; the absorption of VOCs by activated carbon abolished the biocontrol activity of *C. intermedia* 410 [16]. Two strains of *A. pullulans* (L1 and L8) have been reported to produce VOCs to inhibit the growth and infection of postharvest pathogens, including *B. cinerea*, *Colletotrichum acutatum*, and *Penicillium* spp. [66]. Moreover, VOCs have been reported to suppress the mycelial growth, sporulation, and ochratoxin A biosynthesis of *Aspergillus carbonarius* and *Aspergillus ochraceus* [67,68]. VOCs are considered to be potential biological fumigants because of their volatility, which allows them to control postharvest decay without direct contact with the edible commodities. Contarino et al. found that the main VOCs emitted by common antagonistic yeasts include ethyl alcohol, phenylethyl alcohol, 3-methyl-1-butanol, ethyl acetate, and isoamyl acetate [69]. However, VOCs produced by *Muscodor albus* have been reported to cause DNA damage and cytotoxicity in bacterial cells, indicating that some VOCs may be toxic [70]. Therefore, the safety of VOCs should be thoroughly evaluated in future studies.

Several toxins have been reported to be able to control postharvest pathogens, and proteinaceous killer toxins are the most prominent antifungal toxins produced by yeasts [10]. Killer toxins provide a competitive advantage to yeasts, and they can kill fungi (including other yeasts) by a variety of mechanisms, including hydrolyzation of the cell wall, destruction of the cell structure, and inhibition of DNA synthesis [71]. Yeast strains with a particular killer phenotype are immune to their own killer toxins and those in the same class while being lethal to other yeast strains [71]. Owing to this characteristic, killer toxins have long been used in the wine industry to control spoilage yeasts. As natural antifungal proteins, killer toxins are environmentally friendly, nontoxic to mammals, have a good acid tolerance, and have a low probability of inducing resistance. Therefore, killer toxins have been proposed as potential biocontrol agents. Killer toxins produced by *Wickerhamomyces anomalus* BS91 are encoded by the genes *WaEXG1* and *WaEXG2*, showed exoglucanase activity, and associated with biocontrol capabilities against *B. cinerea*, *P. digitatum*, *P. italicum*, *Monilinia fructigena*, and *M. fructicola* [40,72,73,74,75]. *P. membranaefaciens* was found to produce killer toxins PMKT and PMKT2 that target (1→6)-β-D-glucans and mannoproteins in pathogen cell walls and thereby inhibit the growth of postharvest pathogens [76]. Moreover, killer toxins produced by *Debaryomyces hansenii* have been reported to suppress human pathogenic *Candida* yeasts, but only within a certain temperature and pH range, indicating the influence of environmental factors on the antifungal activity of killer toxins [77]. Furthermore, the effects of yeast killer toxins on beneficial microorganisms need to be further evaluated, especially regarding microorganisms in the phyllosphere, on edible commodities, and in the human gut.

## 4. Constraints on the Application of Antagonistic Yeasts, Improvement of Their Biocontrol Efficacy, and Commercial Application

### 4.1. Constraints on the Application of Antagonistic Yeasts

Over the past few decades, numerous yeasts with antifungal properties have been identified, but only a few have been developed as commercial antifungal products. This has mainly been due to the fact that besides having excellent biocontrol efficacy, for commercial application, an antagonistic yeast needs to meet additional requirements. Many commercial factors restrict the development and commercialization of antagonistic yeasts, including the immature commercialization technology, high development costs, small postharvest market, and low market acceptance [8,78]. Furthermore, as the utilization of antagonistic yeasts to control postharvest decay is an emerging industry, the research on antagonistic yeasts remains insufficient. In particular, although many studies have reported on the biocontrol mechanisms of antagonistic yeasts, the specific mechanisms require further clarification.

Biosafety is one of the main reasons for using antagonistic yeasts instead of chemical fungicides. Most of the identified antagonistic yeasts have been directly isolated from the surface of fruits, and humans are already exposed to these yeasts when they eat fresh fruits and vegetables in their daily lives, so there is often less concern about the biosafety of antagonistic yeasts. However, some yeasts may be the origin of human infection under rare circumstances [79,80,81]. Therefore, the biosafety of antagonistic yeasts, including their safety related to skin irritation and ingestion, needs to be fully evaluated. Registration is also an obstacle to the commercialization of many antagonistic yeasts. Biocontrol agents must be approved by relevant regulatory agencies before commercial application. Compared with synthetic chemical fungicides, the registration of an antagonistic yeast is less costly and time-consuming, but it is still a factor to be considered in the development process. The registration of an antagonistic yeast requires an accredited safety assessment report and biocontrol efficacy data. Furthermore, the difficulty of registration varies in different regions. For example, the registration of biocontrol agents in the United States takes an average of 2 years, while in Europe, it takes about 7 years [6]. In China, with government incentives, the registration of biocontrol agents takes about 2–3 years.

Compared with chemical fungicides, antagonistic yeasts still need to be improved in many respects, which also limits their commercialization and market acceptance. Antagonistic yeasts are more expensive than chemical fungicides and are inconvenient to use. Moreover, an ideal biocontrol agent for controlling postharvest decay of fruits and vegetables must be highly effective (>95%) [31]. However, according to the reported researches so far, the biocontrol efficacy using antagonistic yeast alone cannot reach the level demonstrated by chemical fungicides. In addition, the biocontrol efficacy of antagonistic yeasts regarding postharvest decay depends on the high activity and reproductive capacity of the yeasts. In addition, there are issues associated with the use of many antagonistic yeasts, such as their unstable antifungal activity, short shelf life, and strict required storage conditions.

### 4.2. Improvement of the Biocontrol Efficacy

As mentioned above, the use of antagonistic yeast alone to prevent postharvest decay is generally inferior to the use of chemical fungicides. Therefore, while identifying new high-efficacy yeast strains, researchers are also constantly searching for effective ways to strengthen the biocontrol efficacy of existing antagonistic yeasts. The combined use of biological control and physical or chemical methods is an effective way to improve the biocontrol efficacy. For example, hot water treatment (HWT) by immersing fruit in a circulating water bath at 42 °C for 40 min improved the biocontrol efficacy of the antagonistic yeasts *C. guilliermondii* and *P. membranaefaciens* without affecting their growth [82].

Salicylic acid (SA) is an important hormone in plants that is related to the induction of the plant response against pathogens [54]. Qin et al. found that SA treatment increased the antagonism of *R. glutinis* against *P. expansum* and *A. alternata* in sweet cherry fruits [83]. SA at low concentrations increased the activities of defense-related enzymes but had little effect on the growth of the yeast and the two pathogens. This indicated that the biocontrol efficacy enhanced by SA may be related to the triggering of host resistance. The ability of SA to enhance the biocontrol efficacy of biocontrol microbes has been demonstrated in many yeast species [84,85,86]. Methyl jasmonate (MeJA) is another phytohormone that can induce host defense responses [74]. MeJA has also been reported to improve the biocontrol effects of antagonistic yeasts [87,88].

Moreover, it has been reported that exogenous application of brassinosteroids or nitric oxide can induce plant host resistance [89,90], but their synergistic effects when used with antagonistic yeasts remain to be studied. Many natural plant extracts can inhibit the growth and development of pathogenic fungi, such as methyl thujate [91,92], hinokitiol [93], and cinnamic acid [94]. Li et al. reported that cinnamic acid improved the biocontrol efficacy of *C. laurentii* [95], which indicates the potential of combined application of natural plant extracts with antagonistic yeasts for controlling postharvest pathogens. Several other microbial metabolites, such as epsilon-polylysine, natamycin, and rapamycin, have been reported to control postharvest pathogens [96,97,98], and the combined application of microbial metabolites with antagonistic yeasts is worth exploring.

The use of certain chemical reagents or other antifungal methods can also enhance the biocontrol efficacy of antagonistic yeasts. For example, CaCl_2_ has been reported to enhance the efficacy of antagonistic yeasts [99,100,101]. Additionally, chitosan has antifungal properties and can induce host defense responses, and multiple studies have shown that chitosan can enhance the biocontrol efficacy of antagonistic yeasts such as *C. saitoana* [102], *C. laurentii* [103], and *P. membranaefaciens* [104]. Furthermore, inorganic salts (e.g., ammonium molybdate, sodium bicarbonate, and trisodium phosphate) [105,106,107], minerals (e.g., silicon and boron) [108,109], and sugar protectants (e.g., maltose and lactose) [110] have been reported to enhance the biocontrol efficacy of antagonistic yeasts. It has also been reported that the use of a combination of an antagonistic yeast and a low-dose chemical fungicide can achieve a similar biocontrol efficacy to the use of the fungicide alone at a commercial dosage, which is considered to be an effective method to reduce fungicide use [14].

The mixed application of various antagonistic yeasts is also considered to be an effective way to broaden the antifungal spectrum of biocontrol reagents and to enhance the biocontrol efficacy. Calvo et al. found that the combined application of *R. glutinis* and *C. laurentii* improved their ability to control gray mold on apples [111]. However, it should be noted that compatibility between mixed antagonistic yeasts is necessary to ensure that their normal growth and function are maintained. Moreover, Zhao et al. reported that the heterologous expression of flagellin in *S. cerevisiae* significantly induced resistance in the host plant and improved the biocontrol efficacy of the yeast against *B. cinerea*, which suggests that the heterologous expression of elicitors in yeasts may be an effective strategy to improve the biocontrol efficacy [112].

### 4.3. Commercial Application

The commercialization of an antagonistic yeast is a long and costly process requiring extensive testing of toxicology and biocontrol efficacy under commercial conditions. Encouragingly, over the past few decades, a few antagonistic yeasts have been developed and commercialized (Table 1). Aspire (based on *C. oleophila*) and YieldPlus (based on *C. albidus*) are the first-generation commercial antagonistic yeasts [27]. They were available on the market for several years, but they have now been withdrawn due to reasons such as difficulties in market development, low profitability, and inconsistent and low efficacy under commercial conditions [29]. After that, Nexy (another product based on *C. oleophila*) was developed for controlling decay on pome, citrus, and banana, and it was approved for registration throughout the European Union in 2013. Shemer (based on *M. fructicola*) was originally registered in Israel and was successfully used for managing pre- and postharvest diseases on various fruits and vegetables [113]. It was subsequently acquired by Bayer CropScience (Germany) and then sublicensed to Koppert Biological Systems (the Netherlands) to expand its sales [114]. Moreover, Bio-ferm, an Austrian company, developed two products based on *A. pullulans* strains DSM 14940 and DSM 14941, Blossom Protect (Boni-Protect) and Botector. With the mode of action of competition for nutrients and space, Blossom Protect is used to control postharvest decay caused by several fungal pathogens in pome fruit, while Botector is mainly used against gray mold in grape, strawberry, and tomato.

## 5. Conclusions and Perspectives

The environmental pollution and health hazards caused by chemical fungicides have attracted increasing attention from regulatory agencies and consumers, and there is now global interest in reducing or eliminating the use of chemical fungicides. As a potential substitute for chemical fungicides, antagonistic yeasts have been extensively studied over the past few decades, and considerable progress has been made regarding the identification and development of antagonistic yeasts. However, so far, the use of antagonistic yeast alone is still insufficient to completely replace chemical fungicides. There remain many aspects of antagonistic yeasts that could be improved, even for the few commercially available antagonistic yeasts.

Although the application of antagonistic yeasts is limited by many obstacles, there is still great potential for their improvement and development. Due to the regulatory restrictions on chemical fungicides and the declining consumer acceptance of them, it is foreseeable that the use of chemical fungicides will be gradually decreased or even discontinued. The reduction in available products on the market and the increasing demand for safe and effective antifungal products provide opportunities for the development of antagonistic yeast products. The biocontrol efficacy of antagonistic yeasts could be further improved in the future through a variety of strategies, such as combining an antagonistic yeast with a chemical or physical treatment, using multiple antagonistic yeasts, and genetically altering antagonistic yeasts. Moreover, the advancement of molecular biotechnologies and the emergence of “omics” technologies are providing powerful tools for the development and application of antagonistic yeasts.

To promote the commercial application of antagonistic yeasts, efforts can be made in the following aspects: (a) the full verification of biosafety; (b) the in-depth exploration of the involved mechanisms of action; (c) the enhancement and maintenance of biocontrol efficacy under commercial conditions; (d) the development of broad-spectrum antifungal products; (e) the extension of shelf-life; (f) the control of cost and the development of the market; and (g) the understanding of the complex interactions between the components of the biocontrol system, including the antagonistic yeast, pathogen, host, natural microbiome, and environment. Furthermore, gene editing has been considered to be a potentially effective strategy to improve the performance of antagonistic yeasts, though genetically modified microorganisms (GMOs) are restricted due to government policies and low consumer acceptance at present.

## Figures and Tables

**Table 1:** Antagonistic yeast-based commercial products developed for the management of postharvest pathogens (adapted from [9] and [115] with modification).

Product	Yeast	Fruit	Target Pathogens	Manufacturer	In Use
**Aspire**	*Candida oleophila*	Stone fruit, pome, citrus, strawberry	*Botrytis, Penicillium, Monilinia*	Ecogen, USA	No
**Blossom Protect**	*Aureobasidium pullulans*	Pome	*Penicillium, Botrytis, Monilinia*	Bio-ferm, Austria	Yes
**Botector**	*Aureobasidium pullulans*	Grape, strawberry and tomato	*Botrytis cinerea*	Bio-ferm, Austria	Yes
**Candifruit**	*Candida sake*	Pome	*Penicillium, Botrytis, Rhizopus*	IRTA/Sipcam-Inagra, Spain	No
**Nexy**	*Candida oleophila*	Pome, banana, citrus	*Botrytis, Penicillium*	Lesaffre, Belgium	Yes
**Noli**	*Metschnikowia fructicola*	Strawberry, blueberry, grape, stone fruit	*Botrytis,* *Monilinia*	Koppert, The Netherlands	Yes
**Remeo**	*Saccharomyces cerevisiae*	Grape	*Botrytis, Erysiphe,* *Plasmopara*	BASF/Agrauxine, France	Yes
**Shemer**	*Metschnikowia fructicola*	Pome, strawberry, grape, stone fruit	*Botrytis, Penicillium, Rhizopus,* *Aspergillus*	Bayer/Koppert, The Netherlands	Yes
**YieldPlus**	*Cryptococcus albidus*	Pome, citrus	*Botrytis, Penicillium, Mucor*	Lallem, South Africa	No
